# Exploring Patterns of Self-Harm in Autistic Adults Using the Card Sort Task for Self-Harm

**DOI:** 10.1177/13623613261447926

**Published:** 2026-06-08

**Authors:** Mirabel Pelton, Victoria Newell, Blandine French, Ruth Wadman, Ellen Townsend, Sarah Cassidy

**Affiliations:** 1 University of Cambridge, UK; 2 University of Nottingham, UK; 3 University of Bradford, UK

**Keywords:** anxiety, autism, Card Sort Task for Self-Harm, emotional distress, self-harm, suicide

## Abstract

Autistic adults self-harm more often than non-autistic adults, but there are no tools to assess patterns of self-harm in autistic people. This study aimed to (1) review the accessibility of a novel visual task – the Card Sort Task for Self-Harm (CaTS) – to explore self-harm with autistic adults and (2) to pilot the CaTS to identify proximal and distal contributing factors leading to self-harm and experiences following self-harm. First, autistic adults (*n* = 5) with lived experience of self-harm reviewed the CaTS for clarity and accessibility. Second, we undertook a pilot administration of the CaTS and used sequence analysis to explore significant transitions between factors before and after self-harm. Participants (*n* = 29, autistic or possibly autistic, female = 82%, mean age = 41.62) selected, on average, 42 cards to describe self-harm. Most frequently selected cards described agitation (*n* = 25), mental pain (*n* = 22) and depression (*n* = 22), while most infrequently collected cards included being in a gang and talking to a teacher (*n* = 0). Agitation and acting on impulse preceded self-harm. Feeling better, worse, exhausted and hopeless followed self-harm. Improving emotion regulation and meeting autistic communication needs could reduce the transition to self-harm. The CaTS offers a systematic approach to explore self-harm in autistic adults.

**Lay Abstract:**

**Why did we do this study?**

Autistic adults self-harm more often than people who are not autistic. This is particularly worrying because self-harm is something that can contribute to suicide. We know that autistic people feel that their experience of self-harm is not well understood by other people, such as doctors.

**What did we want to know?**

We wanted to know if the card sort task for self-harm (called the CaTS for short) is a useful way to explore self-harm with autistic adults.

**What did we do?**

First, we worked with five autistic adults to make sure the CaTS is clear and works for autistic people. Second, we invited autistic and non-autistic adults to do the CaTS. To *do* the CATS, someone chooses the cards that are relevant to their experience and puts them on a timeline to explain what self-harm is like for them.

**What did we find?**

We found that (1) the CaTS is helpful and accessible to explore self-harm with autistic adults and (2) the CaTS uncovered patterns of self-harm. Twenty-nine U.K.-based autistic adults did the CaTS: most were female (82%, average age was around 42). Participants picked, on average, 42 cards to describe self-harm. The cards that were chosen most often described agitation, mental pain and depression. The cards that were chosen least described being in a gang and talking to a teacher. The order of the cards suggested that people felt agitated and acted impulsively before self-harm. After self-harm, they felt better, worse and hopeless. We found that it is safe and feasible to do the CaTS with autistic people. Support could consider how best to support people who are impulsive and how to prevent people accessing the means to hurt themselves.

## Introduction

Autistic adults^1^ (adults diagnosed with autism spectrum disorder) are at least three times more likely to engage in self-harm (any self-injury or self-poisoning regardless of suicidal intent; Hawton et al., 2012) than non-autistic adults (Blanchard et al., 2021). Self-harm is one of the strongest predictors of suicidal behaviour and death by suicide among autistic people (Cassidy et al., 2022; Moseley et al., 2020). Autistic people are also at increased risk of death by self-harm (regardless of intent), compared to non-autistic people (Cassidy et al., 2022; Hirvikoski et al., 2016; Santomauro et al., 2024). Despite this increased risk, autistic people report poor experiences of seeking support for self-harm, including being disbelieved in their distress or being told that self-harm and mental health difficulties are core autistic characteristics (Camm-Crosbie et al., 2019; Marsden et al., 2024). Within autism research, self-harm has been previously conceptualised as a challenging and/or restricted behaviour characteristic of autism and often associated with co-occurring intellectual disability (ID; Minshawi et al., 2014), which has led to autistic people being excluded from mainstream self-harm research (e.g., Haw et al., 2001; Townsend et al., 2016). However, emerging research has now firmly established the high prevalence of non-suicidal self-injury (NSSI) in autistic people without ID (e.g., Maddox et al., 2017; Moseley et al., 2019; Schwartzman et al., 2023). Despite the prevalence and risk, there remains a lack of evidence exploring the dynamic processes of autistic people’s self-harm, which is needed to inform appropriate treatment and support for this group (Cassidy et al., 2020).

One limitation to taking forward research to describe self-harm in autistic adults is that there are no measures or tools currently developed or validated to assess self-harm among autistic people (Newell et al., 2024), and existing self-harm assessment tools are not considered appropriate or acceptable for autistic adults who self-harm (Newell et al., 2025). Questionnaires designed to assess self-harm in non-autistic people can create response difficulties for autistic people, including unclear questions, lack of meaningful response options and missing important aspects of, or types of, self-harm (Gordon et al., 2025; Newell et al., 2025). Autism-specific experiences of self-harm include meltdown (complete loss of physical and mental control, lashing out at objects, self or others) or stimming (repetitive self-stimulatory behaviour to reduce stress; Marsden et al., 2024). Intentional self-harm, more akin to self-harm in the general population, may have its roots in autism stigma, such as self-punishments for being less than human or ‘not normal’ (Marsden et al., 2024). Autistic characteristics, such as sensory processing differences or co-occurring conditions (such as alexithymia, dissociation and depression), may also impact or influence the experience of self-harm for autistic people (Moseley et al., 2019). Although important contributions to the field, these findings fall short of describing the dynamic processes that lead to self-harm in autistic adults that will allow the development of targeted interventions to support autistic people.

The Card Sort Task for Self-harm (CaTS), originally co-produced with young people, is a research tool rooted in self-harm literature and drawing on the ‘ideation-to-action’ suicide theories. Constructs include thwarted belongingness and perceived burdensomeness, and suicidal capability originating from the Interpersonal Theory of Suicide (Joiner, 2005) and defeat and entrapment from the Integrated Motivational Volitional model (O’Connor, 2011). The CaTS explores the complexity and dynamics of self-harm by visually mapping the transitions between factors leading to an episode of self-harm, and following self-harm, allowing the process to be systematically described across individuals (Townsend et al., 2016; Wadman et al., 2017). The task may be particularly appropriate to autistic thinking and communication styles for several reasons. First, given well-documented differences in social communication among autistic people, the CaTS uses visual cue cards consisting of written words, that support communication. Second, written cards cues can support autistic people who experience alexithymia (difficulty describing emotional states) to describe their emotions. Third, the CaTS can be tailored to a particular group to capture a range of relevant factors, such as looked-after young people (Wadman et al., 2017), LGBTQIA+ (Bird et al., 2025) and prison populations (Keatley, 2018) or to compare antecedents across groups (i.e., young adults compared to older adults Lockwood et al., 2023). This is particularly useful in capturing the potentially unique experience of self-harm in autistic adults, which will likely require adapted assessment and prevention strategies (Cassidy & Rodgers, 2017). Overall, this suggests that the CaTS may be a promising tool for exploring and understanding self-harm in autistic adults.

The current study sets out to (1) review the accessibility of the CaTS as a tool for exploring self-harm with autistic adults and (2) pilot the CaTS with autistic adults to make a preliminary exploration of proximal and distal contributing factors, antecedents and sequiturs of self-harm.

## Method

### Participants

In Phase 1, lived experience collaborators were five autistic adults with lived experience of self-harm (see community involvement statement). Participants in Stage 2 were 29 U.K.-based autistic adults aged 18–73 years (mean age 42 years, 24 female). Inclusion criteria for Phase 2 were (1) being adult (age = >18); (2) being autistic (diagnosed or self-identifying) or possibly autistic (awaiting diagnosis) and (3) having experienced self-harm in the last 6 months. Participants were recruited from the Autistica Discover Network (U.K.-based autism research charity), MQ Mental Health Research and Reddit. We also aimed to recruit a comparison group of non-autistic adults. Recruitment began in January 2020 and was suspended due to the COVID-19 pandemic in March 2020, and then restarted in January 2022 until August 2022.

Sixty-one participants (31 autistic and 30 non-autistic) gave consent to take part in the study. Four consented participants did not proceed with the CaTS due to being unable to find a convenient time (*n* = 2) or withdrew due to wellbeing (*n* = 2). Participant records were removed prior to analysis from participants who (1) participated prior to the pandemic (*n* = 2); (2) misrepresented their lived experience, presumably for financial gain and took part multiple times (*n* = 19, see French et al., 2024); (3) followed an incorrect procedure (*n* = 1); and (4) scored below the cutoff for autism on the Comprehensive Autistic Trait Inventory (CATI; *n* = 4). The small number of non-autistic participants (*n* = 4) meant we were unable to proceed with planned group comparisons. In line with research reporting that suicidal thoughts and behaviours develop equivalently for those with possible versus confirmed autism (Cassidy et al., 2022; Pelton et al., 2023), we proceeded with analysis for the 29 autistic or possibly autistic participants.

Thus, 29 participants were retained in the analysis (*n* = 24 formal diagnosis of autism, *n* = 1 awaiting diagnosis and *n* = 4 scoring over cutoff on the CATI indicating possible undiagnosed autism). Of these, 26 participants reported current mental health diagnoses including anxiety (*n* = 18), depression (*n* = 18), anorexia (*n* = 3), bulimia (*n* = 1), obsessive-compulsive disorder (OCD; *n* = 2), complex/post-traumatic stress disorder (*n* = 5) and borderline/emotional unstable personality disorder (*n* = 6). Twelve participants reported additional conditions including chronic fatigue, attention deficit disorder, Tourette’s syndrome and functional neurological disorder. Mean CATI score for the sample was 157.72 (range = 107–180). Two participants diagnosed with autism scored below cutoff (<134) on the CATI. All participants identified as White British.

## Measures

### Demographic Information

Participants self-reported age, ethnicity, mental health and neurodevelopmental diagnoses. Self-reported autism diagnosis status options included not autistic, possibly autistic – awaiting diagnosis, possibly autistic – not awaiting diagnosis, or autistic. Online self-report shows good concordance with diagnostic tests in validation studies and allows for online recruitment of vulnerable groups (Fombonne et al., 2022; Nock et al., 2008).

### Autistic Characteristics

Autistic characteristics were measured using the CATI (English et al., 2021). The CATI is a 42-item self-report measure of autistic traits that reports dimensions of social interactions, communication, social camouflage, cognitive rigidity, repetitive behaviours and sensory sensitivity. Total scores range from 42 to 210 with a score of 134 indicating possible autism.

### CaTS

The CaTS (Townsend et al., 2016; Wadman et al., 2017) consists of 117 cards with thoughts, feelings, events, behaviours and self-harm supports/services printed on them. These include items such as *I could not tell anyone how I was feeling, I felt very anxious* or *I was very agitated and restless*. Seven cards describe experiences after an episode of self-harm, such as *I felt better after self-harm* or *A and E*^2^ *staff were caring and understanding*. There are time point cards ranging from *Longer than Six Months* to *Afterwards* to guide participants to identify the time at which an experience contributes to self-harm. The full list of CaTS items is provided in supplementary information (Supplemental Table S1).

Participants indicated their emotional state using a visual analogue scale (VAS) with a 1–10 scale before and after undertaking the CaTS. This was used to ensure individual participant safety and wellbeing after completing the task and also to assess the overall safety of autistic adults completing the CaTS online (see section Phase 2: Undertaking a pilot administration of the CaTS).

### Community Involvement Statement

This study has at its heart the lived experience of autistic people and addresses a topic of high importance. Phase 1 was a community engagement phase to assess the views of autistic adults about the feasibility of using the CaTS to explore self-harm and to seek their advice on suggested adaptations for using CaTS with autistic adults. When Phase 2 recommenced following the U.K. lockdown, the group proposed moving the CaTS online using MIRO (an online digital whiteboard) to display, select and present the cards in an online meeting with a researcher. They undertook a pilot of the online CaTS and suggested changes to the procedure to ensure this was safe and accessible for autistic people.

### Phase 1: Adapting the CaTS With and for Autistic People

In Phase 1, we recruited five autistic adults with lived experience of self-harm in December 2019, who advised on the appropriateness of the CaTS for autistic adults. They reviewed participant-facing materials and CaTS task instructions to ensure these were clear. They also reviewed the cards to ensure these were clear and relevant and explored whether new autism-specific cards needed to be added. The group suggested that a ‘longer than 6 months’ card needed to be added to the timeline before ‘6 months before’, given that many distal life experiences could relate to an episode of self-harm. There was not a strong consensus on the inclusion of specific new cards and concern that adding new cards may be overwhelming for participants. Therefore, we instead planned to explore whether any themes or new categories emerged from the new spontaneous cards written by autistic participants when completing the CaTS.

### Phase 2: Undertaking a Pilot Administration of the CaTS

In 2020, participants attended the university and undertook the CaTS using physical cards, as in Townsend et al. (2016). From January 2022, participants received an information sheet and the opportunity to ask questions before giving informed consent online and completing a survey of demographics and the CATI. An initial wellbeing meeting determined individual accessibility requirements including whether they preferred to undertake the CaTS online or at the university. All participants opted to take the CaTS online, in which case, we invited them to share their technological preference, such as whether they whether they planned to use a phone, tablet or computer and whether they wished to use a set of physical cards alongside the online platform. These details were recorded in a wellbeing plan tailored from https://sites.google.com/view/mentalhealthinautism/resources/tools. Ethical approval was obtained from the University of Nottingham School of Psychology Ethics Committee. Data are highly sensitive and are available upon reasonable request to Dr Sarah Cassidy.

**Figure 1.**
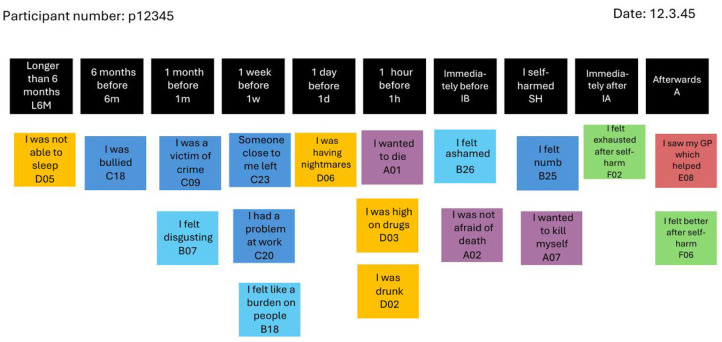
Illustrates a representation of a fictional participant’s completed MIRO board. *Note.* Black cards across the top mark time points from distal events (‘longer than six months before’) to the self-harm event (‘I self-harmed’) and beyond. Before starting, cards are grouped around the board’s edge by category: dark blue = events, red = support, yellow = behaviour, light blue = emotions, green = afterwards. The participant selects relevant cards and arranges them under the time points, either navigating the MIRO board themselves or communicating choices to a researcher, possibly using physical cards.

Participants completed the CaTS in a second appointment. Participants were invited to think of their most recent experience of self-harm, and then choose cards that were relevant to their recent experience. Participants placed cards where relevant along the timeline and then organised the cards according to importance within each time point. Participants could use as many cards as appropriate and were able to write their own cards if they felt the provided cards did not fully capture their experience. Once complete, their card selection was downloaded from MIRO or, where physical cards were used, a photo was taken of their card selection, and the card sequence was recorded in a code character string (see Figure 1).

Participants rated their current emotional state (‘How are you feeling?’) on a VAS at the start and end of the session. Some participants reported finding that assessment tools were over-simplistic or challenging; thus, participants were asked to consider how they felt at that moment, with respect to how they normally feel, and the researcher recorded any other information participants wanted to describe how they felt. Where participants reported a drop in mood, a researcher remained with the participant and followed their personalised action plan as set out in their wellbeing plan until their mood improved.

### Analysis Strategy

Handwritten cards were not included in the quantitative analysis, as these cards had not been consistently available to all participants via the initial set of cards provided. Review of handwritten cards did not reveal a consistent theme and tended to be very specific and unique to the individual (see Supplementary 3). Frequency analysis found the most frequently chosen cards by participants, the mean number of cards chosen at each time point and the category of cards chosen by each participant at each time point. Lag sequential analysis following the procedure established by Townsend et al. (2016) was used to explore whether there was a significant sequential structure in the data. Lag sequential analysis explores the frequency of each possible discrete two-card sequence (antecedent-sequitur pairing) in the frequency-filtered sequences. For example, how often did *I felt very anxious* occur before *I felt agitated and restless*? Card sequences are recorded in a transition frequency matrix, and the most significant important transitions within the pattern structure of the data are identified by the largest adjusted residuals, indicating significant interdependence between two factors. We ascertained high-, medium-, and low-frequency cards using cutoffs determined by identifying distinctive changes in the gradient of the slope of the card frequencies using a diagram like a scree plot – that is, where the plot changed in gradient indicated the cutoff. To limit the number of items included in the sequence analysis, high-frequency items plus pooled ‘medium-frequency’ and ‘low-frequency’ items were included, along with the time stamps ‘6 months before’ and ‘I self-harmed’. This ensures a manageable number of items for inclusion in the transitional frequency matrix and state transition diagram. Lag sequence analysis identified sequential patterns in the data. Cutoffs of adjusted residuals (<1.9) reduced the number of transitions to be meaningfully representable in a state transition diagram. We additionally report Yules Q and transformed kappa for the strongest transitions in line with recommendations for lag sequential analysis (O’Connor, 1999). All analyses were conducted in R (version 4.4.1; R core team 2024). We used the r package *GrpString* to produce the frequency matrix and the r package *lagSequential* to produce the chi-square analysis as a statistical test of cross-dependency and to adjust residuals. The state transition diagram was rendered in *qGraph* using the Fruchter–Rheingold algorithm that places highly connected nodes at the centre. R script is contained in the supplementary information.

## Results

### Frequency Analysis

**Figure 2.**
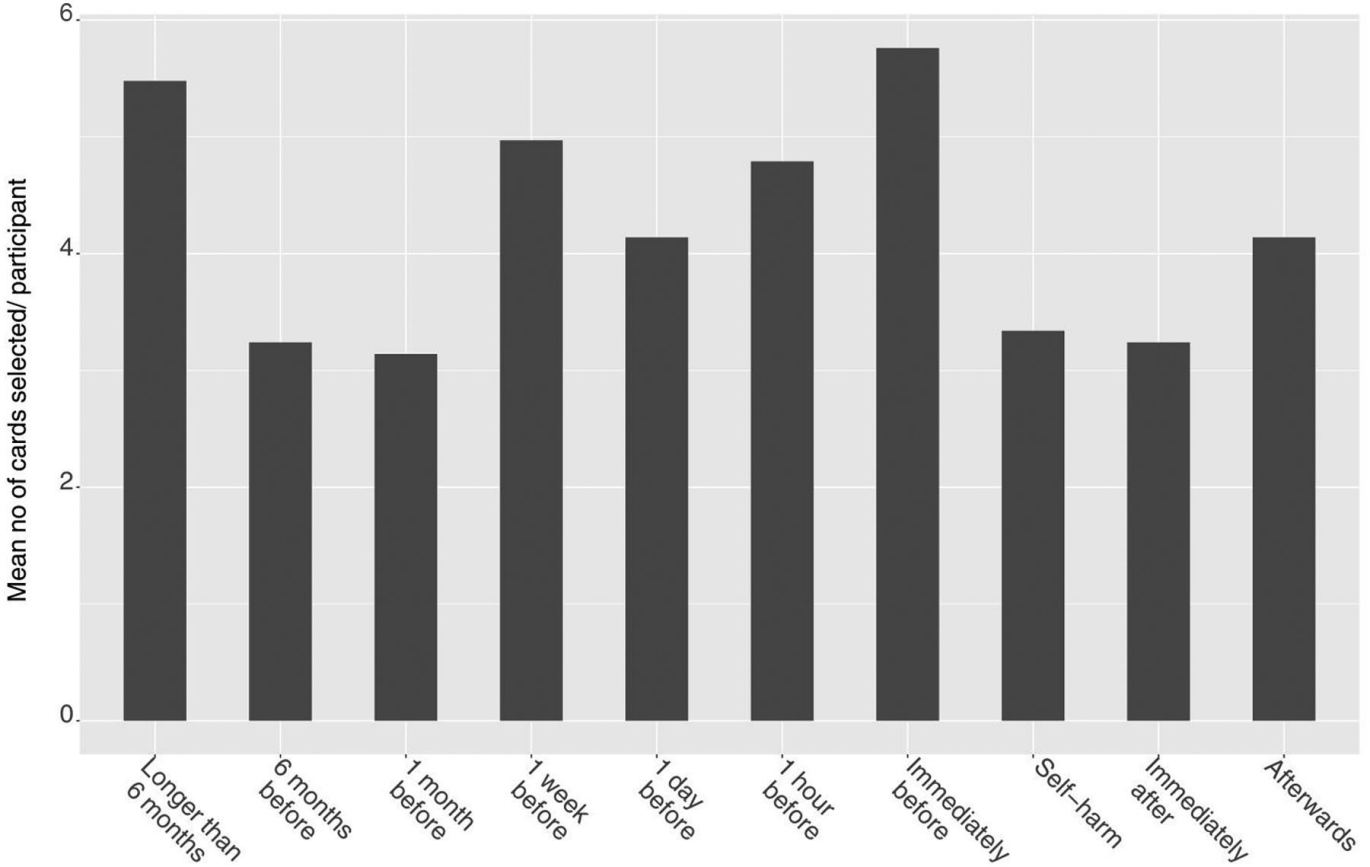
Bar chart showing the mean number of cards selected per participant at each time point. *Note.* Figure 2 shows the mean number of cards selected per participant at each time point from the period longer than 6 months before self-harm, to the point of self-harm and afterwards.

Participants used 42 cards on average to complete the CaTS. As shown in Figure 2, participants chose more cards (*M* = 5.5) to describe the period longer than 6 months before self-harm, fewer to describe 6 months (*M* = 3.2) or 1 month before (*M* = 3.1). The largest number of cards (*M* = 5.8) was used immediately before the point of self-harm. Fewer cards were used to describe the period immediately after the point of self-harm (*M* = 3.2).

**Figure 3.**
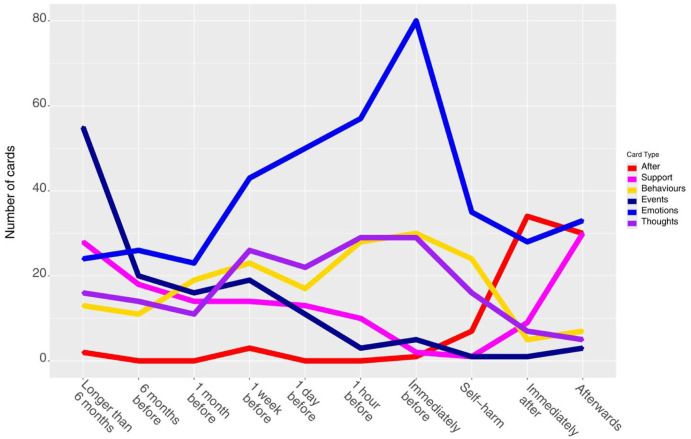
Line graph showing the frequency of cards selected at each time point based grouped by type of card. *Note.* Figure 2 shows the total number of cards selected at each time point broken down into groups (colours indicated in the legend indicate the type of card represented by each colour) from the period longer than 6 months before self-harm, to the point of self-harm and afterwards.

As shown in Figure 3, across the data set, event cards were the most frequently selected cards to describe the period longer than 6 months before self-harm (*n* = 55), while emotion cards were the most frequently selected at the point immediately before self-harm (*n* = 80).

### Cards Added to the CaTS by Autistic People

Autistic people wrote 145 additional cards (shown in Supplementary 3). Some of these duplicated or clarified existing cards (such as the ‘paramedics were friendly and understanding’ rather than ‘A and E staff were friendly and understanding’), while others added cards which described autism-specific themes, such as unmet autism and mental health support needs, poor understanding of or poor support for autism. Most experiences were described by only one participant; except *I felt calm* and *I felt relieved* which were added two participants. Thus, no handwritten cards met the threshold for anything other than low-frequency cards and were, thus, not included in the sequence analysis.

### High-Frequency Cards

As shown in Table 1, the most frequently chosen cards were: *I was very agitated and restless (n* *=* *25), I felt exhausted (n* *=* *23)* and *the mental pain was unbearable, I felt depressed and sad* and *I hated myself (n* *=* *22 each)*. Cards not chosen by any participants were *I was taken into foster care, I had unprotected sex, I got involved with a gang, I got into trouble at school/work* and *I talked to a teacher which helped* (*n* = 0 each).

**Table 1.** High-Frequency Cards Selected by Autistic Adults to Describe Their Experience of Self-Harm.

Card code	Card wording	Frequency/*n* = 29 (%)
D01	I was very agitated and restless	25 (86.21)
B08	I felt exhausted	23 (79.31)
B03	The mental pain was unbearable	22 (75.86)
B04	I felt depressed and sad	22 (75.86)
B10	I hated myself	22 (75.86)
B16	I felt I could not escape from feelings or situations	21 (72.41)
B17	I felt like a burden on people	21 (72.41)
A09	I could not tell anyone how I was feeling	19 (65.52)
B05	I felt very anxious	19 (65.52)
B14	I felt trapped	19 (65.52)
B18	I felt very hopeless about the future	19 (65.52)
D14	I had access to the means to hurt myself	19 (65.52)
D15	I did it on impulse without planning	19 (65.52)
F02	I felt worse after self-harm	19 (65.52)
F06	I felt better after self-harm	19 (65.52)
B06	I felt worthless	18 (62.07)

### Sequence Analysis

The chi-square test indicated that the observed frequency of two-factor transitions was significantly different to that expected by chance [χ^2^(361) = 470.02, *p* < .01]. Thus, there was a significant (non-random) sequential structure in the transitional frequency matrix.

**Figure 4.**
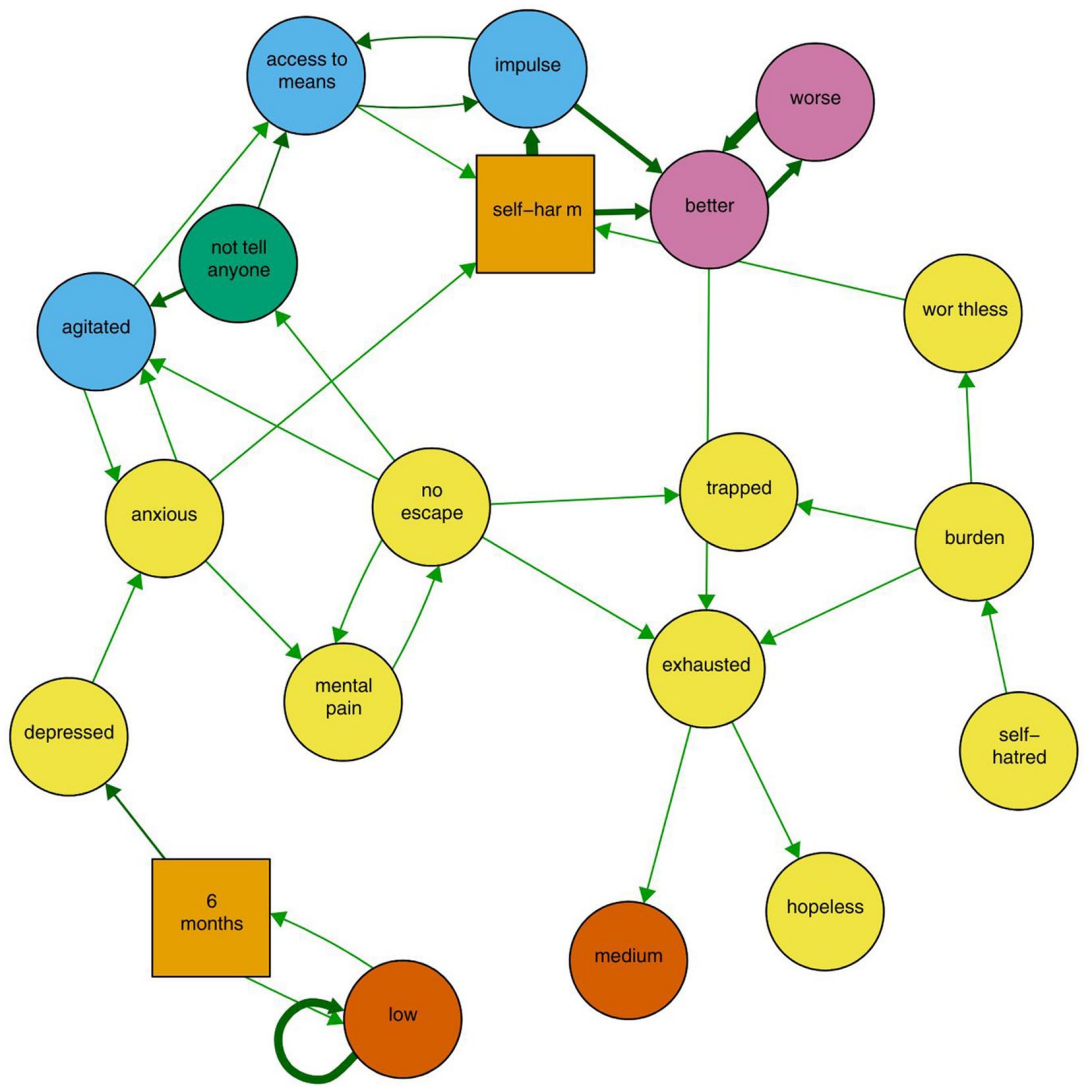
State transition diagram showing significant transitions in the most recent episode of self-harm for autistic adults. *Note.* 1. Width of arrows indicates relative strength of transition. 2. Key: yellow = emotions, green = thoughts, dark orange = pooled items, blue = behaviours, pink = afterwards, orange squares = time points. 3. See Table 2 for individual transition strength. 4. See Table 1 for full card wording.

As shown in Figure 4 and Table 2, the strongest transition was from *I self-harmed* to *I did it on impulse and without planning*, which also inter-connected with *I had access to the means to hurt myself*. There were strong bi-directional transitions between: (1) *I did it on impulse without planning* and *I had access to the means to hurt myself* and (2) *I felt better after self-harm* and *I felt worse after self-harm*. Other notable transitions include *I was unable to tell anyone how I was feeling* which preceded both *I was very agitated and restless*, and *I had access to the means to hurt myself. I felt like I could not escape from feelings or situations* preceded *I could not tell anyone how I was feeling. I felt better after self-harm* preceded *I felt exhausted* which preceded *I felt very hopeless. Low-frequency items preceded low-frequency items* and *6* *months before.*

**Table 2.** Transitions between High-Frequency Cards for Most Recent Episode of Self-Harm with Adjusted Residuals >1.9.

Antecedent node label (card code)	Sequitur node (card code)	Adjusted residual
I self-harmed ([SH])	I did it on impulse without planning (D15)	7.97
I felt worse after self-harm (F02)	I felt better after self-harm (F06)	6.75
Low-frequency cards (LF)	Low-frequency cards (LF)	6.24
I felt better after self-harm (F06)	I felt worse after self-harm (F02)	5.1
I self-harmed ([SH])	I felt better after self-harm (F06)	5.07
I did it on impulse without planning (D15)	I felt better after self-harm (F06)	4.76
I could not tell anyone what I was feeling (A09)	I felt agitated and restless (D01)	4
6 months ago ([6M])	I felt depressed and sad (B04)	3.27
I could not tell anyone what I was feeling (A09)	I had access to the means to hurt myself (D14)	2.98
I did it on impulse without planning (D15)	I had access to the means to hurt myself (D14)	2.98
I had access to the means to hurt myself (D14)	I did it on impulse without planning (D15)	2.98
I felt like a burden on people (B17)	I felt worthless (B06)	2.89
I felt worthless (B06)	I felt exhausted (B08)	2.82
I felt I could not escape from feelings or situations (B16)	I could not tell anyone what I was feeling (A09)	2.78
I felt I could not escape from feelings or situations (B16)	I felt trapped (B14)	2.78
I felt like a burden on people (B17)	I felt trapped (B14)	2.78
I felt exhausted (B08)	I felt very hopeless about the future (B18)	2.78
I felt very anxious (B05)	The mental pain was unbearable (B03)	2.69
I felt depressed and sad (B04)	I felt very anxious (B05)	2.69
I felt I could not escape from feelings or situations (B16)	The mental pain was unbearable (B03)	2.5
The mental pain was unbearable (B03)	I felt I could not escape from feelings or situations (B16)	2.5
I hated myself (B10)	I felt like a burden on people (B17)	2.5
I felt exhausted (B08)	Medium-frequency items (MF)	2.46
I felt agitated and restless (D01)	I felt very anxious (B05)	2.45
I felt agitated and restless (D01)	I had access to the means to hurt myself (D14)	2.45
I felt worthless	I self-harmed ([SH])	2.27
I felt I could not escape from feelings or situations (B16)	I felt agitated and restless (D01)	2.26
I felt very anxious (B05)	I self-harmed ([SH])	2.18
I had access to the means to hurt myself (D14)	I self-harmed ([SH])	2.18
6 months ago (6M)	Low-frequency items (LF)	2.15
Low-frequency items (LF)	6 months ago (6M)	2.14

*Note.* Table 2 lists significant transitions in descending order with the preceding card ‘antecedent’ displayed in the left-hand column, following card ‘sequitur’ in the middle and adjusted residuals in the right-hand column. Larger residuals indicate stronger transitions. Figure 3 shows a graphical representation of these transitions. As described in the results, cards include high-frequency cards (indicated with alphanumeric code [e.g., B05]), grouped items for low-/medium-frequency cards (LF/MF) and retained time points (SH and 6M).

### Emotional State

There was no significant difference between mean scores on the VAS before (*M* = 6.71, *SD* = 2.27) and after (*M* = 6.41, *SD* = 2.13) undertaking the CaTS [*t*(27) = 1.03, *p* = .3].

## Discussion

We explored the acceptability of the CaTS with autistic people and undertook a pilot administration to identify proximal and distal contributing factors, antecedents and sequiturs to self-harm. Our lived-experience collaborators confirmed the safety and acceptability of the CaTS for autistic participants. This was also confirmed by participants, all of whom were able to complete the CaTS, with no overall significant difference in participants’ mood before compared to after completing the task. Participants who completed the CaTS identified wide-ranging thoughts, feelings and behaviours that contributed to self-harm, of which the most common were feeling agitated and restless, exhausted, depressed and experiencing unbearable mental pain. Negative life events were distal to self-harm, while emotional distress was proximal. Negative life events, over 6 months prior to self-harm, contributed to depression. Anxiety preceded unbearable mental pain and agitation. Negative emotions (burdensomeness, worthlessness and entrapment) and having no one to talk to preceded having access to means and acting on impulse which preceded self-harm. Overall, this study makes a significant contribution to research on self-harm in autistic adults by demonstrating the acceptability of a novel task and providing the first pilot data describing dynamic antecedents and sequiturs of self-harm in autistic adults.

Findings suggest the CaTS was adapted to be clear and accessible for autistic adults, reflecting the work of other studies that positive co-production can develop safe, accessible research methods to explore sensitive topics such as self-harm, suicide and mental health (Nicolaidis et al., 2020; Rodgers et al., 2023). Diverse negative life events were distal to self-harm, in line with findings in the general population (Cleare et al., 2018; Madge et al., 2011) and studies reporting childhood trauma as a contributing factor to self-harm among autistic people with and without formal diagnosis (Carmassi et al., 2019; Warrier & Baron-Cohen, 2021). A peak of negative emotional experiences immediately prior to self-harm reflects conceptual research describing that self-harm serves to regulate overwhelming distressing emotions in the general population (Chapman et al., 2006) and has been cited as a possible contributor to self-harm in autistic people (Dell’Osso et al., 2023). Most consistently reported experiences among autistic adults were feeling agitated and restless, experiencing unbearable mental pain, depression and self-hatred. These are perhaps unsurprising, given that among autistic people, depression is one of the most frequently diagnosed mental health difficulties (Lever & Geurts, 2016), and agitation and self-hatred have previously been associated with self-harm and suicidal ideation (Camm-Crosbie et al., 2019; Moseley et al., 2019, 2020; Pelton et al., 2023). These frequently selected experiences are similar to those reported in samples of general population young people (Lockwood et al., 2023; Townsend et al., 2016), with agitation and restlessness possibly featuring more prominently in our autistic sample. Notably, the high endorsement of agitation, restlessness may act as proxies for overwhelm, such as meltdown or burnout, which share characteristics of overwhelming mental distress (Mantzalas et al., 2024; Phung et al., 2021). Cards not selected by any participants were those experiences specifically related to young people, such as being in school (Townsend et al., 2016), and suggest these cards could be removed to ensure a more relevant starter set for adult participants. Overall, our findings support and describe experiences of extreme distress and existing self-harm risk factors among autistic adults.

Our findings extend previous research by putative descriptions of *how* specific experiences might build towards self-harm for autistic adults. Burdensomeness, worthlessness, entrapment and arrested flight preceded having no one to talk to (Campos et al., 2022; Townsend et al., 2016; Wadman et al., 2017). Worthlessness and dehumanisation are well-documented in the literature on autistic mental health distress and may be significant in autistic depression, but poorly understood by others (Bitsika & Sharpley, 2023; Cage et al., 2019). Our finding that ‘having no one to talk to’ preceded self-harm is significant, given significant previous findings that ‘being unheard’ is a significant experience for autistic people who self-harm (Marsden et al., 2024), in line with research that autistic people report not being taken seriously when they present at services with self-harm, mental health difficulties and suicidality (Camm-Crosbie et al., 2019).

These findings have important potential interpretations to be explored in future research to inform future support for self-harm in autistic people. Our findings could also indicate loneliness and having no one to disclose difficult feelings to (Cassidy et al., 2021c; Causton-Theoharis et al., 2009; Pelton et al., 2020), meaning an absence of protective social connectedness which might potentially disrupt the transition to self-harm. Our findings could also be interpreted to suggest habituation to masking autistic traits and characteristics to fit in with social situations, which could impede disclosure and access to support (Cassidy et al., 2019; Mitchell et al., 2021). Future research could explore whether applying frameworks such as the ‘Double Empathy Problem’ (Milton, 2012) – which posits that communication breakdowns occur due to reciprocal misunderstandings between autistic and non-autistic people, rather than solely the result of autistic social communication difficulties – could promote greater inclusivity of autistic communication styles in clinical settings (Culpin et al., 2017) and reduce pressure to mask (Mitchell et al., 2021), thereby supporting authentic opportunities to be heard. Overall, this reinforces the importance of social support (Tham et al., 2020; Townsend et al., 2016) in recovery from self-harm as in the general population and suggests that understanding and responding to autistic communication styles could be an important priority for future clinically focussed research.

Having no one to talk to preceded having access to means, which interacted with acting on impulse without planning. This reflects research in young people (including looked-after young people) using the CaTS, which highlights that for many groups, removing access to means and providing strategies to reduce impulsivity could disrupt the transition to self-harm (Lockwood et al., 2023; Townsend et al., 2016). Unusually, we report that self-harm preceded acting on impulse; however, this finding reflects our participants’ view that the experience of self-harm was *characterised by*, rather than *preceded*, or *followed by*, impulsivity. The wording and administration of this card could be considered by future studies. Importantly, having no one to talk to also preceded agitation and restlessness. This could suggest that movement provides an alternative way of coping in place of seeking access to means to self-harm, reflecting research describing how repetitive movement and self-stimulatory behaviours (‘stimming’) play an important role in emotion regulation for autistic people (Kapp et al., 2019). Future research could explore the role of movement in coping for autistic people and test this pathway in a matched sample of autistic and non-autistic people to infer its relevance as an autism-specific intervention target.

Self-harm was followed by feeling better, which interacted with feeling worse. This reflects well-documented complex emotions associated with self-harm and aligns with suicide theory, such as the Interpersonal Theory of Suicide (Joiner, 2005). The temporary relief of emotional distress after self-harm highlights the reinforcement mechanism that can contribute to the development of acquired capability for suicide (Moseley et al., 2022), followed by the subsequent return of hopelessness. Moreover, feeling better after self-harm preceded feeling exhausted. This sequence also resonates with chronic exhaustion as a core characteristic of autistic burnout (Raymaker et al., 2020), where burnout has been proposed as a risk factor for self-harm among autistic people (Forcey-Rodriguez, 2023; Stewart et al., 2022). Exhaustion is also reported to be of relatively greater importance in the development of suicidality in autistic compared to non-autistic people (Pelton et al., 2023). Feeling exhausted preceded hopelessness, in line with research that feeling more exhausted than usual is an indicator of depression in autistic people (Cassidy et al., 2021a). Overall, our data suggest a putative chain in which the energy cost of the self-harm act leads to exhaustion, in turn precipitating hopelessness, aligning with research showing that, over time, self-harm can contribute to increased suicidality.

These findings identify modifiable intervention targets that may be of relevance to autistic people. Ongoing initiatives to reduce anxiety and depression may be important to reduce self-harm by avoiding the build-up of unbearable mental distress (El Baou et al., 2023; White et al., 2018). Promoting autonomy and self-worth may arrest the development of feelings of entrapment and defeat, which could constitute pertinent indicators for clinical assessment (Joiner et al., 2009). Providing autistic people with opportunities to express their experiences, guided by principles of ‘double empathy’ (autistic and non-autistic communication styles are equally valid) (Milton, 2012) and reducing pressure to mask (Mitchell et al., 2021) may constitute an important aspect of support for self-harm for autistic people. Strategies such as reducing access to means that are perhaps more established in the general population should be considered in the specific context of self-harm among autistic people (Blanchard et al., 2021; Lai et al., 2023). The CaTS is currently being co-developed and evaluated as a clinical assessment tool in the general population (Lockwood et al., 2025). Taken in sum, these measures could reduce the incidence of self-harm among autistic people.

Future research will extend these findings by undertaking qualitative analyses of the handwritten cards to identify autism-specific cards that could be added to the CaTS starter set. Future studies could extend this pilot study to a larger sample of autistic adults, including those with intersectional identities, young autistic people or autistic people with ID, who may be at increased or different risk. Research could also take as its starting point the lived experience of self-harm among autistic adults to ensure that all meaningful and important experiences are included in the CaTS. These steps would enable clinically focussed research to produce guidance on *how* best to understand and intervene at specific points in the sequence to develop tailored support for autistic people who self-harm. This could include understanding more about the role of movement or stimming in relation to coping with mental health distress to develop novel therapeutic or coping options.

### Strengths and Limitations

This is the first study to adapt and apply a novel task to understand the dynamic factors contributing to self-harm with and for autistic adults. This study was co-designed with autistic adults and addresses a high-priority concern of the autism community (Cassidy et al., 2021b). These findings should be considered in the context of limitations; our sample is not representative of the autism community given it is largely female with a mean age of 42, so findings should not be generalised across the autism spectrum or to other high-risk groups such as adolescents and transition-aged youth. However, this could reflect that autistic women are at increased risk of self-harm (Hull et al., 2024) and that gendered assumptions about self-harm could lead to under-reporting of self-harm in men in general (Lutz et al., 2023). This could also reflect research reporting that self-harm may continue into middle and older adulthood for people with high autistic traits (Stewart et al., 2022). Future research could consider applying the CaTS in other priority groups, such as young people, and should prioritise ensuring representation from autistic people with intersectional identities and could consider whether pictorial or icon prompts might increase accessibility.

We were unable to undertake planned comparisons between autistic and non-autistic people, given that small sample that remained after removing unreliable data, and those with high self-reported autistic traits indicating possible autism. Future research should apply our recommendations for recruiting reliable online participants (French et al., 2024) and could extend these findings to compare the experiences of those with high autistic traits and without diagnosis, as they may constitute a priority group given absent support and challenges with self-identity (Cassidy et al., 2014; Stagg & Belcher, 2019).

## Conclusions

This study demonstrates that the CaTS is an accessible tool for autistic adults to explore self-harm. The unique contribution of the study lies in moving beyond prevalence and static risk factors to illuminate, for the first time, pathways of dynamic antecedents and sequiturs of self-harm in autistic adults. By visualising the dynamic sequence of self-harm, we can identify potential intervention points. Future research should seek to understand autism-specific indicators of and coping strategies used in place of self-harm. Support should better meet autistic communication styles, promote autonomy and self-worth and reduce access to means.

## Supplemental Material

sj-docx-1-aut-10.1177_13623613261447926 – Supplemental material for Exploring Patterns of Self-Harm in Autistic Adults Using the Card Sort Task for Self-Harm Supplemental material, sj-docx-1-aut-10.1177_13623613261447926 for Exploring Patterns of Self-Harm in Autistic Adults Using the Card Sort Task for Self-Harm by Mirabel Pelton, Victoria Newell, Blandine French, Ruth Wadman, Ellen Townsend and Sarah Cassidy in Autism

sj-docx-2-aut-10.1177_13623613261447926 – Supplemental material for Exploring Patterns of Self-Harm in Autistic Adults Using the Card Sort Task for Self-Harm Supplemental material, sj-docx-2-aut-10.1177_13623613261447926 for Exploring Patterns of Self-Harm in Autistic Adults Using the Card Sort Task for Self-Harm by Mirabel Pelton, Victoria Newell, Blandine French, Ruth Wadman, Ellen Townsend and Sarah Cassidy in Autism

sj-docx-3-aut-10.1177_13623613261447926 – Supplemental material for Exploring Patterns of Self-Harm in Autistic Adults Using the Card Sort Task for Self-Harm Supplemental material, sj-docx-3-aut-10.1177_13623613261447926 for Exploring Patterns of Self-Harm in Autistic Adults Using the Card Sort Task for Self-Harm by Mirabel Pelton, Victoria Newell, Blandine French, Ruth Wadman, Ellen Townsend and Sarah Cassidy in Autism

sj-pdf-5-aut-10.1177_13623613261447926 – Supplemental material for Exploring Patterns of Self-Harm in Autistic Adults Using the Card Sort Task for Self-Harm Supplemental material, sj-pdf-5-aut-10.1177_13623613261447926 for Exploring Patterns of Self-Harm in Autistic Adults Using the Card Sort Task for Self-Harm by Mirabel Pelton, Victoria Newell, Blandine French, Ruth Wadman, Ellen Townsend and Sarah Cassidy in Autism

sj-R-4-aut-10.1177_13623613261447926 – Supplemental material for Exploring Patterns of Self-Harm in Autistic Adults Using the Card Sort Task for Self-Harm Supplemental material, sj-R-4-aut-10.1177_13623613261447926 for Exploring Patterns of Self-Harm in Autistic Adults Using the Card Sort Task for Self-Harm by Mirabel Pelton, Victoria Newell, Blandine French, Ruth Wadman, Ellen Townsend and Sarah Cassidy in Autism

## Notes

1. We use identity-first language (autistic people) throughout to reflect the published preference of the autistic community worldwide (Keating et al., 2023; Kenny et al., 2016). We acknowledge a range choices exist, and language choices evolve over time.

2. ‘Accident and Emergency’ (commonly termed ‘A and E’ in British English for the department of a hospital dealing with emergency presentations, also termed Emergency Department).
