# Supplementary material for: Exploring Patterns of Self-Harm in Autistic Adults Using the Card Sort Task for Self-Harm
Source: Autism. 2026 Jun 8;30(7):1802–15. doi: 10.1177/13623613261447926 (PMC13287349; doi:10.1177/13623613261447926)
Supplement: sj-docx-1-aut-10.1177_13623613261447926 – Supplemental material for Exploring Patterns of Self-Harm in Autistic Adults Using the Card Sort Task for Self-Harm [file sj-docx-1-aut-10.1177_13623613261447926.docx]

Supplementary table 1: Card Sort Task for Self-harm card items

Thoughts

| A01 | I wanted to die |
| --- | --- |
| A02 | I was not afraid of death |
| A03 | There was no one to turn to for help |
| A04 | I could not trust anyone |
| A05 | I struggled to make decisions |
| A06 | I could not think of anything else to do |
| A07 | I wanted to kill myself |
| A08 | I could not solve a problem I faced |
| A09 | I could not tell anyone how I was feeling |
| A10 | No one listened to me or took me seriously |
| A11 | I thought about being very badly treated as a young child |
| A12 | I trusted a caregiver |
| A13 | I had flashbacks about something bad that happened |

Feelings

| B01 | I was angry |
| --- | --- |
| B02 | I felt I would not be able to change myself in the future |
| B03 | The mental pain was unbearable |
| B04 | I felt depressed and sad |
| B05 | I felt very anxious |
| B06 | I felt worthless |
| B07 | I felt disgusting |
| B08 | I felt exhausted |
| B09 | I felt energized |
| B10 | I hated myself |
| B11 | I felt hopeful about the future |
| B12 | I felt I could change for the better in the future |
| B13 | I felt I could not change for the better in the future |
| B14 | I felt trapped |
| B15 | I felt defeated |
| B16 | I felt I could not escape from feelings or situations |
| B17 | I felt like a burden on people |
| B18 | I felt very hopeless about the future |
| B19 | I felt humiliated |
| B20 | I felt like I did not belong |
| B21 | I felt ignored |
| B22 | I felt rejected |
| B23 | I felt abandoned |
| B24 | I did not know what I was feeling |
| B25 | I felt numb |
| B26 | I felt ashamed |

Events

| C01 | I was being abused physically |
| --- | --- |
| C02 | I was being abused mentally |
| C03 | I had an argument with my friend |
| C04 | I had an argument with my boyfriend/girlfriend |
| C05 | I had an argument with my parent/caregiver |
| C06 | I got into trouble with the police |
| C07 | I was raped |
| C08 | I knew someone who was self-harming |
| C09 | I was a victim of a crime |
| C10 | Someone I knew killed themselves |
| C11 | Lots of people I knew were doing it |
| C12 | I read about self-harm on the internet |
| C13 | I discussed self-harm in a forum on the internet |
| C14 | I was rejected by my parents |
| C15 | I was taken into foster care |
| C16 | I was taken into a residential care home. |
| C17 | I received no support from caregivers. |
| C18 | I was bullied |
| C19 | I had a problem at school |
| C20 | I had a problem at work |
| C21 | Someone close to me died |
| C22 | I moved to a different home |
| C23 | Someone close to me left |
| C24 | I moved to a different school |
| C25 | I was having problems in a close relationship |
| C26 | I had a problem at university/college |
| C27 | Someone I knew tried to kill themselves |
| C28 | My home life was not very good |

Behaviour

| D01 | I was very agitated and restless |
| --- | --- |
| D02 | I was drunk |
| D03 | I was high on drugs |
| D04 | I had unprotected sex |
| D05 | I was not able to sleep |
| D06 | I was having nightmares |
| D07 | I got involved with a gang |
| D08 | I am insensitive to pain |
| D09 | I was drinking alcohol |
| D10 | I was taking illegal drugs |
| D11 | I planned it carefully |
| D12 | I isolated myself from others |
| D13 | I did other things to hurt myself (starved myself, drank too much) |
| D14 | I had access to the means to hurt myself |
| D15 | I did it on impulse without planning |
| D16 | I got into trouble at school/work |
| D17 | I got into trouble at home |
| D18 | I am used to pain |

Support and services

| E01 | Someone listened to me and took me seriously |
| --- | --- |
| E02 | I received therapy which helped |
| E03 | I received therapy which did not help |
| E04 | I talked to a friend which helped |
| E05 | I talked to a friend which did not help |
| E06 | I talked to my caregivers which helped |
| E07 | I talked to my caregivers which did not help |
| E08 | I saw my GP which helped |
| E09 | I saw my GP which did not help |
| E10 | I phoned a helpline which helped |
| E11 | I phoned a helpline which did not help |
| E12 | I went to counselling which helped |
| E13 | I went to counselling which did not help |
| E14 | I read a self-help book – it helped |
| E15 | I read a self-help book – it did not help. |
| E16 | I talked to a teacher which helped |
| E17 | I talked to a teacher which did not help |
| E18 | I talked to a social worker which helped |
| E19 | I talked to a social worker which did not help |
| E20 | I received help and support from a user-led service (e.g. Harmless) |
| E21 | I depended on a caregiver for help and support |
| E22 | I talked to a boyfriend/girlfriend which helped |
| E23 | I talked to a boyfriend/girlfriend which did not help |
| E24 | I talked to a mental health support worker which helped |
| E25 | I talked to a mental health support worker which did not help |
| F01 | I went to hospital for overdose or self-injury |
| F02 | I felt worse after self-harm |
| F03 | Self-harm stopped me from killing myself |
| F04 | A & E staff were friendly and understanding |
| F05 | A & E staff were not friendly and understanding |
| F06 | I felt better after self-harm |
| F07 | I felt no different after self-harm |

Time points

| [6M+] | Longer than 6 months |
| --- | --- |
| [6M] | 6 months before |
| [1M] | 1 month before |
| [1W] | I week before |
| [1D] | I day before |
| [1H] | 1 hour before |
| [IB] | Immediately before |
| [JB] OR [SH] | I self-harmed |
| [IA] | Immediately after |
| [A] | Afterwards |
