# Supplementary material for: Exploring Patterns of Self-Harm in Autistic Adults Using the Card Sort Task for Self-Harm
Source: Autism. 2026 Jun 8;30(7):1802–15. doi: 10.1177/13623613261447926 (PMC13287349; doi:10.1177/13623613261447926)
Supplement: sj-docx-2-aut-10.1177_13623613261447926 – Supplemental material for Exploring Patterns of Self-Harm in Autistic Adults Using the Card Sort Task for Self-Harm [file sj-docx-2-aut-10.1177_13623613261447926.docx]

Supplementary 2: Card frequency usage

| **Card code** | **Card wording** | **Frequency** |
| --- | --- | --- |
| D01 | I was very agitated and restless | 25 |
| B03 | The mental pain was unbearable | 22 |
| B04 | I felt depressed and sad | 22 |
| B10 | I hated myself | 22 |
| B08 | I felt exhausted | 21 |
| B17 | I felt like a burden on people | 21 |
| A09 | I could not tell anyone how I was feeling | 19 |
| B14 | I felt trapped | 19 |
| B16 | I felt I could not escape from feelings or situations | 19 |
| B18 | I felt very hopeless about the future | 19 |
| D14 | I had access to the means to hurt myself | 19 |
| D15 | I did it on impulse without planning | 19 |
| F02 | I felt worse after self-harm | 19 |
| F06 | I felt better after self-harm | 19 |
| B05 | I felt very anxious | 18 |
| B06 | I felt worthless | 18 |
| A05 | I struggled to make decisions | 17 |
| A06 | I could not think of anything else to do | 17 |
| A08 | I could not solve a problem I faced | 17 |
| B15 | I felt defeated | 17 |
| B26 | I felt ashamed | 17 |
| D12 | I isolated myself from others | 17 |
| B01 | I was angry | 16 |
| B24 | I did not know what I was feeling | 16 |
| D05 | I was not able to sleep | 16 |
| A13 | I had flashbacks about something bad that happened | 15 |
| B22 | I felt rejected | 15 |
| D06 | I was having nightmares | 15 |
| A01 | I wanted to die | 14 |
| A07 | I wanted to kill myself | 14 |
| B23 | I felt abandoned | 14 |
| B25 | I felt numb | 14 |
| D13 | I did other things to hurt myself (starved myself, drank too much) | 14 |
| D18 | I am used to pain | 14 |
| E04 | I talked to a friend which helped | 14 |
| A04 | I could not trust anyone | 13 |
| B21 | I felt ignored | 13 |
| F03 | Self-harm stopped me from killing myself | 13 |
| A10 | No one listened to me or took me seriously | 12 |
| B02 | I felt I would not be able to change myself in the future | 12 |
| B20 | I felt like I did not belong | 12 |
| E01 | Someone listened to me and took me seriously | 12 |
| C02 | I was being abused mentally | 11 |
| D08 | I am insensitive to pain | 11 |
| A02 | I was not afraid of death | 10 |
| A03 | There was no one to turn to for help | 10 |
| B13 | I felt I could not change for the better in the future | 10 |
| B19 | I felt humiliated | 10 |
| C18 | I was bullied | 10 |
| A11 | I thought about being very badly treated as a young child | 9 |
| C20 | I had a problem at work | 9 |
| C21 | Someone close to me died | 9 |
| B07 | I felt disgusting | 8 |
| C25 | I was having problems in a close relationship | 8 |
| C28 | My home life was not very good | 8 |
| D11 | I planned it carefully | 8 |
| E11 | I phoned a helpline which did not help | 8 |
| F01 | I went to hospital for overdose or self-injury | 8 |
| C04 | I had an argument with my boyfriend/girlfriend | 7 |
| E05 | I talked to a friend which did not help | 7 |
| E09 | I saw my GP which did not help | 7 |
| E12 | I went to counselling which helped | 7 |
| F04 | A & E staff were friendly and understanding | 7 |
| B09 | I felt energized | 6 |
| B12 | I felt I could change for the better in the future | 6 |
| C07 | I was raped | 6 |
| C14 | I was rejected by my parents | 6 |
| C23 | Someone close to me left | 6 |
| E02 | I received therapy which helped | 6 |
| E08 | I saw my GP which helped | 6 |
| E22 | I talked to a boyfriend/girlfriend which helped | 6 |
| E24 | I talked to a mental health support worker which helped | 6 |
| E25 | I talked to a mental health support worker which did not help | 6 |
| C01 | I was being abused physically | 5 |
| C17 | I received no support from caregivers. | 5 |
| D09 | I was drinking alcohol | 5 |
| E03 | I received therapy which did not help | 5 |
| E07 | I talked to my caregivers which did not help | 5 |
| E10 | I phoned a helpline which helped | 5 |
| E20 | I received help and support from a user-led service (e.g. Harmless) | 5 |
| E21 | I depended on a caregiver for help and support | 5 |
| F05 | A & E staff were not friendly and understanding | 5 |
| F07 | I felt no different after self-harm | 5 |
| C03 | I had an argument with my friend | 4 |
| C05 | I had an argument with my parent/caregiver | 4 |
| C08 | I knew someone who was self-harming | 4 |
| C19 | I had a problem at school | 4 |
| E13 | I went to counselling which did not help | 4 |
| E14 | I read a self-help book, it helped | 4 |
| A12 | I trusted a caregiver | 3 |
| B11 | I felt hopeful about the future | 3 |
| C06 | I got into trouble with the police | 3 |
| C10 | Someone I knew killed themselves | 3 |
| C26 | I had a problem at university/college | 3 |
| C27 | Someone I knew tried to kill themselves | 3 |
| D02 | I was drunk | 3 |
| D03 | I was high on drugs | 3 |
| D17 | I got into trouble at home | 3 |
| E06 | I talked to my caregivers which helped | 3 |
| E15 | I read a self-help book, it did not help. | 3 |
| E18 | I talked to a social worker which helped | 3 |
| E19 | I talked to a social worker which did not help | 3 |
| E23 | I talked to a boyfriend/girlfriend which did not help | 3 |
| C09 | I was a victim of a crime | 2 |
| C12 | I read about self-harm on the internet | 2 |
| C13 | I discussed self-harm in a forum on the internet | 2 |
| C22 | I moved to a different home | 2 |
| E17 | I talked to a teacher which did not help | 2 |
| C11 | Lots of people I knew were doing it | 1 |
| C16 | I was taken into a residential care home. | 1 |
| C24 | I moved to a different school | 1 |
| D10 | I was taking illegal drugs | 1 |
| C15 | I was taken into foster care | 0 |
| D04 | I had unprotected sex | 0 |
| D07 | I got involved with a gang | 0 |
| D16 | I got into trouble at school/work | 0 |
| E16 | I talked to a teacher which helped | 0 |
