# Supplementary material for: Exploring Patterns of Self-Harm in Autistic Adults Using the Card Sort Task for Self-Harm
Source: Autism. 2026 Jun 8;30(7):1802–15. doi: 10.1177/13623613261447926 (PMC13287349; doi:10.1177/13623613261447926)
Supplement: sj-docx-3-aut-10.1177_13623613261447926 – Supplemental material for Exploring Patterns of Self-Harm in Autistic Adults Using the Card Sort Task for Self-Harm [file sj-docx-3-aut-10.1177_13623613261447926.docx]

**Supplementary 3: Handwritten cards by autistic people**

*Note:* potentially identifying information has been redacted. Acronyms in square brackets expanded by researchers for ease of interpretation.

**Feelings**

I was anxious about seeing a GP [general practitioner, community physician in the UK] about [redacted]

I was feeling a bit calmer

I was feeling over-whelmed

I was frightened

I felt calm^[[1]](#footnote-1)^1

I felt relieved^1^

emotional breakdown"

I feel disconnected and get into the bath and it doesn't feel like me

I feel frustrated because I'm back stuck in this cycle again

I felt defeated and overwhelmed

I felt despairing

I felt disappointed

I felt embarrassed

I felt euphoric

I felt grief

I felt guilty about damaging myself

I felt I could not solve a problem I faced

I felt I was having a manic episode

I felt I would not be able to positively change myself in the future

I felt isolated working from home

I felt like I was not good enough because I couldnt do the thing I should be able to do

I felt like I was trapped

I felt more positive

I felt over whelmed

I felt scared (that my mental health was going down hill again)

I felt there was no one to turn to to help

I felt to blame

I felt ugly

I felt very anxious like it was my fault

I felt worse after self-harm

I felt worse immediately after self- harm

It felt like an OCD [obsessive compulsive disorder] compulsion

My heart was racing

**Behaviours**

I was having more intrusive thoughts about self-harm/suicide again

I was having nightmares

I was having problems managing my OCD compulsions

D03 [refers to card in starter CaTS set] I was high on prescription drugs

Binge eating"

chronic suicidal ideation since I was 6/7"

I am silent"

I barely ate anything because I was stressed"

I blamed myself and want to hurt or punish myself"

I did other things to hurt myself [redacted]

I didn't drink anything. Maybe if I was ill someone would listen.

I dissociated

I fled

I got to hospital to avoid harming myself further

I had tried to ask people to help but they didnt understand

I had not been self caring

I have been self-harming since I was 6 years-old

I have made multiple suicide attempts starting at age 7

I have to do it. It's what I do to keep well

I keep going until it happens again tomorrow

I stopped taking my medication

I struggled to make decisions

I talked to someone who did not take me seriously

I tried to hide it

I tried to hide my feelings to protect others

I trusted my partner

I was salivating

I was stuck indoors

I was superdissociated

I wasn't aware I was doing it

MY brain got stuck

My brain shutdown

People wouldn't notice the change in my behaviours

**Events**

Adult Social Care not tied to mental health service, that make choices over psyhical

[redacted] invalidation by safeguarding services

Annoucement of change in care co-ordinator"

CF [childhood factors]

Being on my own is fearful, no longer conected to cmht [Community Mental Health Team] after long time instionised"

Change in care is being implemented"

Christmas was coming up and my family don't understand autism"

crisis team did no want to get involded with me on the phone and put the phone down on me as my times up. parasuide,"

harassment by neighbour"

[redacted] gaslighting and anti-social behaviour

Found by the police. converned for my welfare than following treadmill with crisi teams bore or half asleep"

disaibilty than pick n chooicing from cmht"

Domestic servant, while being trageted by services"

Emerging memories of past trauma"

I ended up in a psychiatric hospital

I had friends who were feeling ill or having a difficult time

I had my PIP [personal independence payment – UK process for assessing eligibility for disability support payments] tribunal

I had unmet health and autism needs

I lived in a house where there was domestic violence

[redacted] Invalidation in healthcare services

I was sexually assaulted

I was sexually harrassed for three years by the same person

[redacted] discharge from psychiatric hospital

I was involved-in an accident

I worked in a field that was triggering for me

Increased work stress due to COVID [Corona Virus Disease]

Invalidation from family over the years

Locally funded service which did not help

looked at scientific reviews and peer review research papers

[redacted] accident and ill-health of family member

My 2 children lived with me

[redacted] anniversary of significant death

My eldest at 5 told me her dad sexually abused her

My job role became uncertain

My PA [personal assistant] left

My pet died

my physicals health got worse

My workplace did not understand me

No relatives no parents, what family had have their own problems

partner involved in car accident

People don't understand autism.

[redacted] serious safeguarding incident with police involvement

Prolonged sexual abuse

Retrauma from previous problem

Severe anorexia

Statutory services don't help. They said 'you can't have a severe condition if you don't have a learning disability

Supportive co-worker left

Transition from hospital to community

[redacted] domestic crisis

the house was a mess

[redacted to avoid identification] invalidation in healthcare services

the pandemic has caused changes and anxeity for me

There was a lot of stressful events

There was a situation I could not sort out or control

**Thoughts**

I can't control what my body is doing because of menopause

I can't repair it

I cant cope

I could not think of anything else to do

I couldn't control myself

I don't have the energy to fight it off any more

I don't know words to tell someone what has gone wrong and what I was feeling

I dreaded anyone finding out

I had a bit more hope for the future

I had a diagnosis and couldnt get my head round it

I had a lot of different work pressures

I had repetitive thoughts about the situation

I need to know what was real

I wanted to feel something

that looks and feels untidy I'll pick it

The mental pain was difficult (not unbearable)

**Afterwards**

AE [accident and emergency – UK hospital emergency department] made referral for [redacted] rehab

Call 111 [in the UK, 111 is the telephone number for a service run by the public health system providing non-emergency medical advice and referral] and say what I'd done to be made safe from myself"

Could not be dishonest to the police of my location of attempt"

I properly care for my wounds as best I could

I still have chronic suicidal ideation

if it was an attempt I was frustrated I hadn't succeeded

or it was an attempt to kill myself

Physical pain provides a distraction from mental pain, but only for a short time

The overwhelmed feelings felt resolved

This helped the emotional pain for 1 or 2 days

There wasnt intent to do a huge injury, it was to prevent the feelings, but that escaltaed in itself

**Support**

I had autism work coahing which helped me

I wasn't sure what support or services to access

I received support from an occupational therapist

I wrapped myself in a blanket for 20 minutes

My GP listened to me and took me seriously

No therapy or support was offered

1. 1 Chosen by two participants. [↑](#footnote-ref-1)
