# Supplementary figures and images for: Exploring Patterns of Self-Harm in Autistic Adults Using the Card Sort Task for Self-Harm
Source: Autism. 2026 Jun 8;30(7):1802–15. doi: 10.1177/13623613261447926 (PMC13287349; doi:10.1177/13623613261447926)

Participant unique ID: p12345

Date: 12.34.56

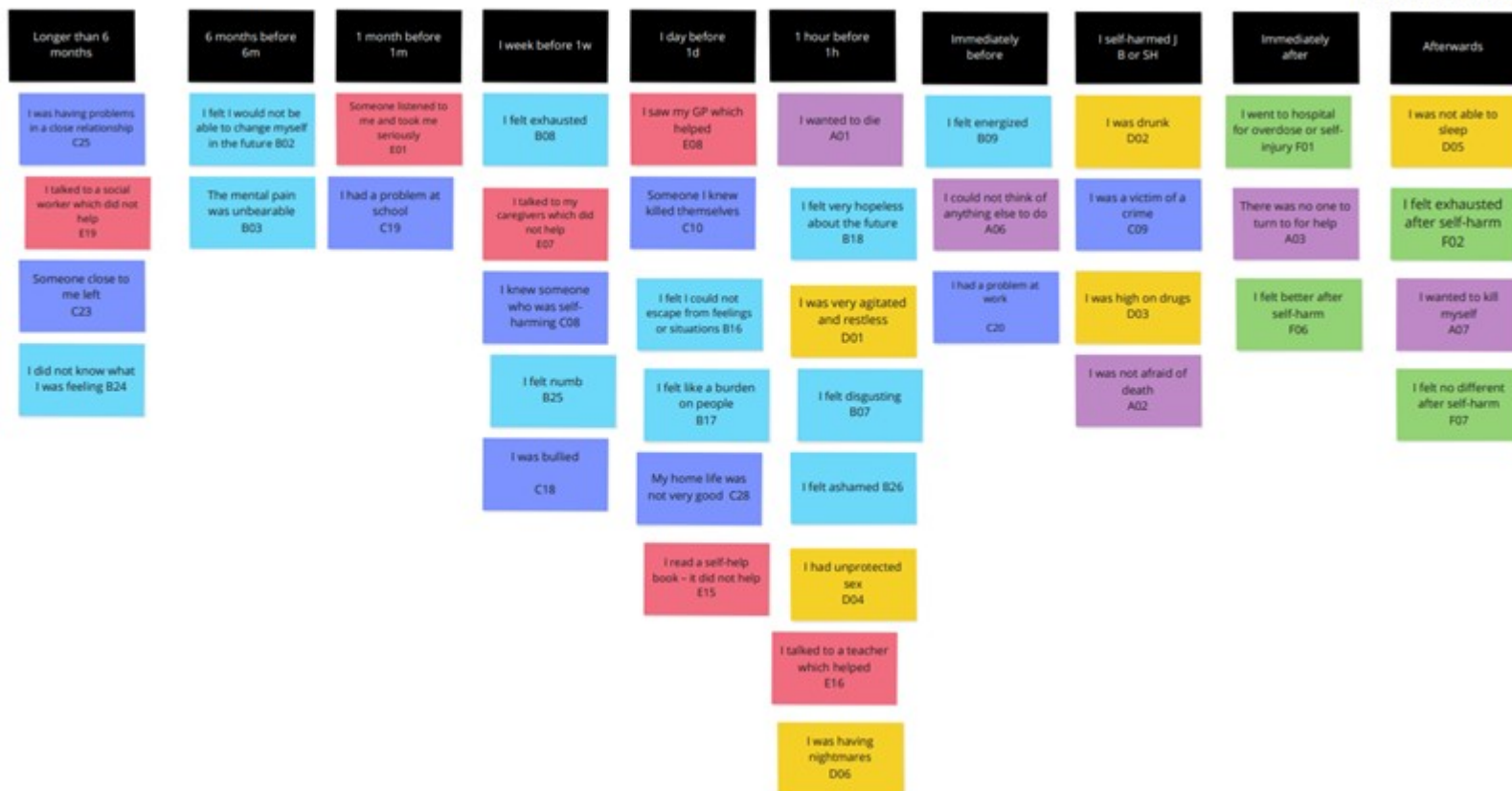

Supplement: sj-pdf-5-aut-10.1177_13623613261447926 – Supplemental material for Exploring Patterns of Self-Harm in Autistic Adults Using the Card Sort Task for Self-Harm [file sj-pdf-5-aut-10.1177_13623613261447926.pdf]
